# Current evidence on hyaluronic acid injections for rotator cuff tendinopathy: A scoping review

**DOI:** 10.1177/17585732251324484

**Published:** 2025-03-13

**Authors:** Alexandre Lavigne, Edmond Sandouk, Andrew Hiett, Min Cheol Chang, Valérie Bélanger, Martin Lamontagne, Michael Khadavi

**Affiliations:** 1 Department of Physical Medicine and Rehabilitation, 25443 Centre hospitalier de l'Université de Montréal, Montreal, QC, Canada; 2 Faculty of Medicine, 25443 Université de Montréal, Montreal, QC, Canada; 3 Department of Physical Medicine and Rehabilitation, University of Kansas Medical Center, Kansas City, KS, USA; 4 Department of Physical Medicine and Rehabilitation, 36707 College of Medicine, Yeungnam University, Daegu, Republic of Korea; 5 Department of Physical Medicine and Rehabilitation, 36896 CHU de Québec-Université Laval, Quebec City, QC, Canada; 6 Kansas City Orthopedic Alliance, Overland Park, KS, USA

**Keywords:** rotator cuff, hyaluronic acid, tendinopathy, shoulder, injection

## Abstract

**Introduction:**

There is growing evidence that hyaluronic acid (HA) injections can significantly improve pain and function in rotator cuff tendinopathy. However, there is no consensus regarding the optimal parameters for HA injections. This narrative review explores the procedural considerations for HA injections in rotator cuff tendinopathy.

**Methods:**

A literature search using Pubmed and Cochrane was conducted to assess procedural considerations for HA injections in rotator cuff tendinopathy including the type of HA (linear vs. cross-linked), the molecular weight (low, moderate, and high), the combination of HA with other products, the number and frequency of injections, the injection guidance, and the adverse effects.

**Results:**

Nine randomized-controlled trials and two prospective non-randomized studies assessed the efficacy of HA injections for rotator cuff tendinopathy, and their characteristics were thoroughly analyzed. Two studies compared the efficacy of different molecular weight HA. One study assessed the efficacy of HA combined with extracorporeal shockwave therapy.

**Conclusion:**

Highlights of the findings include the clinical benefits of HA injections for rotator cuff tendinopathy, the better tolerability of low molecular weight HA compared to high molecular weight, the safer adverse effect profile of HA compared to glucocorticoid injections, and the synergistic effect of HA and extracorporeal shockwave therapy.

## Introduction

Rotator cuff tendinopathy is a highly prevalent and debilitating shoulder pathology.^1–3^ Traditionally, managing rotator cuff tendinopathy begins with education, activity modification, physical therapy, extracorporeal shockwave therapy (ESWT), and oral analgesia if needed,^4,5^ with corticosteroid injections (CSIs) serving as second-line treatment.^6^ However, CSIs have shown increasing evidence for local side effects such as tendon toxicity,^7–9^ in addition to systemic adverse effects such as hyperglycemia, hypertension, adrenal suppression, and transient alterations in bone metabolism.^10–13^

Hyaluronic acid (HA) is another injectable treatment for rotator cuff tendinopathy that has shown promising effects.^14,15^ In-vitro studies have shown that HA injected around the tendon directly contributes to tendon repair by promoting fibroblast activities such as extracellular matrix synthesis and by increasing tenocyte proliferation.^16,17^ HA also inhibits tenocyte apoptosis, while increasing collagen type 1 production, resulting in the restoration of the ideal ratio of Type 1 to Type 3 collagen.^17,18^ Furthermore, HA has proangiogenic properties mainly due to the upregulation of vascular endothelial growth factor expression, contributing to tendon healing.^16,19^

HA is often classified and studied based upon its molecular weight (MW). Three common categories of MW are high (HMW), moderate (MMW), and low (LMW) which correspond to molecular weights of ≥3000, 1500–3000, and ≤1500 kDa, respectively.^20^ Additionally, HA can also be classified as linear or cross-linked based on how HA chains are engineered.^21^ Cross-linked HA forms a network of HA chains and is thought to slow the in vivo HA degradation and possibly improve treatment effects, according to animal studies.^22,23^

This narrative review assesses the following HA parameters that need to be determined when performing HA injections for rotator cuff tendinopathy: type of HA, molecular weight, combination of HA with other products, number of injections, injection guidance, and potential adverse effects. No review article assessing HA for rotator cuff tendinopathy to date has assessed these variables specifically.

## Methods

A literature search using the biomedical search engines Pubmed/Medline and the Cochrane Central Register of Controlled Trials was conducted to gather relevant literature published from inception until September 2024. RCTs and lower-grade evidence prospective studies were analyzed to get the most comprehensive analysis of the current literature. All articles written in English and French were included. Keywords used in the search strategies were [“hyaluronic acid” OR “hyaluronate” OR “viscosupplementation”] AND [“shoulder” OR “rotator cuff” OR “supraspinatus” OR “infraspinatus” OR “subscapularis” OR “teres minor” OR “tendinopathy” OR “tendinitis”]. Studies that were not related to HA and rotator cuff tendinopathy were excluded. The literature search took place from July 2024 until September 2024.

In addition to analyzing the injection characteristics and the clinical outcomes of each included study, the monographs of each injectable medication used in those studies were thoroughly examined.

Study outcomes were deemed favorable when the efficacy of HA for pain and functional improvement was significantly greater than the efficacy of saline or other control treatments. They were judged as “neutral outcomes” when there was not a significant difference between HA's efficacy and the control treatments. Finally, they were considered unfavorable when the efficacy of HA was equal or inferior to the efficacy of saline or inferior to the efficacy of other control treatments.

## Results

The search strategies yielded 337 scientific articles. Among those, there were 11 clinical studies assessing the efficacy of HA injections for rotator cuff tendinopathy. Moreover, two clinical studies compared the efficacy of different molecular weights of HA. All the studies included in this review used linear HA; no study on rotator cuff tendinopathy has used cross-linked HA.

### Characteristics and outcomes of studies assessing the efficacy of HA for rotator cuff tendinopathy

The efficacy of HA in rotator cuff tendinopathy has been studied in nine RCTs and two prospective non-randomized studies. The characteristics of these studies are summarized in Table 1.

**Table 1.** Summary of the hyaluronic acid injection parameters used in each included study on rotator cuff tendinopathy.

Study	Design	Treatment comparison	Sample size	Intervention	Guidance	HA used	Control intervention	Follow-up length	Comparison	Favorable outcome
Cai et al.^24^	RCT	PRP versus HA versus (PRP + HA) versus saline	184 (44 HA; 48 HA + PRP)	Four subacromial injections with 1 week apart	US	4 mL of SH (Haohai Biological Technology, Shanghai, China), unspecified MW	4 mL saline Or 4 mL PRP Or 2 mL of SH + 2 mL of PRP	12 months	(PRP + HA) > PRP > HA > saline	Yes
Kim et al.^25^	RCT	HA versus CSI	80 (38 HA)	Three subacromial injections with 1 week apart versus only 1 CSI, (PT as a cointervention)	US	2 mL of Hyruan plus, 3000 kDa, 20 mg	1 mL (5 mg) of dexamethasone disodium phosphate, 4 mL lidocaine (2%, 20 mg/mL) and 5 mL saline	12 weeks	At 12 weeks, HA > CSI	Yes
Penning et al.^26^	RCT	HA versus CSI versus saline	159 (51 HA)	Three subacromial injections with 3 weeks apart	Landmark	2 mL of HA (Ostenil), 1600 kDa, 8 mL of lidocaine 1%	2 mL triamcinolone acetonide (10 mg/mL), 8 mL lidocaine 1%	26 weeks	At 6 and 12 weeks: CSI and saline > HA. At 26 weeks : HA = saline = CSI	Neutral
Moghtaderi et al.^27^	RCT	HA versus saline	40 (20 HA)	Three subacromial injections with 1 week apart	US	2 mL of Fermathron, 2000 kDa, 20 mg	Saline	12 weeks	HA > saline	Yes
Chou et al.^27^	RCT	HA versus saline	51 (25 HA)	Five subacromial injections with 1 week apart	Landmark	2.5 mL of SH, ARTZ, 1200 kDa, 25 mg	Saline	6 weeks	HA > saline	Yes
Esmaily et al.^29^	RCT	HA versus PT	79 (55 HA)	One subacromial injection	US	20 mg (2 mL) LMW-HA 1% (500–700 kDa, Hyalgan, Fidia Farmaceutici, Abano Terme, Italy) or 20 mg (2 mL) HMW-HA 1% (2000 kDa, Synogel, NikanTeb Kimia Pharmaceutical Co Ltd, Tehran, Iran)	PT	6 months	HA > PT	Yes
Rezasoltani et al.^30^	RCT	HA versus PT	51 (28 HA)	One subacromial injection	US	2 mL (20 mg) LMW HA 1% (500–700 kDa, Hy- algan; FidiaPharma)	PT	12 weeks	HA > PT	Yes
Flores et al.^32^	RCT	(HA + PT) versus PT	84 (42 HA)	Two subacromial injections with 1 week apart	US	40 mg sodium hyaluronate/2 mL, OSTENIL^®^ TENDON, 1600 kDa	PT	90 days	(HA + PT) = PT	Neutral
Ko et al.^31^	RCT	(HA + ESWT) versus HA	93 (93 HA)	Three subacromial injections with 1 week apart	Landmark	ARTZ Dispo, 25 mg of SH, 620–1170 kDa (Seikagaku, Tokyo, Japan)	Same HA, but without ESWT	12 months	HA + ESWT > HA	Yes
Merolla et al.^33^	Prospective non-randomized comparative study	HA versus PT	48 (25 HA)	Two subacromial injections with 1 week apart	US	1.2 mL of LMW (1000 kDa) HA (SportVis, MDT Int’l SA, Switzerland)	PT	24 weeks	HA > PT	Yes
Cipolletta et al.^34^	Prospective observational study	HA + PT	32 (32 HA)	Two subacromial injections with 2 weeks apart	US	12 mg/1.2 mL, 1000 kDa, (Tendovis, MDT Int’l SA, Geneva, Switzerland)	N/A	12 weeks	N/A	Yes

CSI: corticosteroid injection; ESWT: extracorporeal shockwave therapy; HA: hyaluronic acid; HMW: high molecular weight; kDa: kilodalton; LMW: low molecular weight; mg: milligram; mL: milliliter; MW: molecular weight; N/A: not applicable; PRP: platelet-rich plasma; PT: physical therapy; RCT: randomized controlled trial; SH: sodium hyaluronate; US: ultrasound.

Cai et al.^24^ conducted a study on participants with rotator cuff tendinopathy associated with bursal-sided partial thickness supraspinatus tears. Participants received four weekly ultrasound-guided (USG) injections in the subacromial bursa of either platelet-rich plasma (PRP), HA of unspecified molecular weight, saline, or both PRP and HA. Of the 184 participants, 48 received PRP plus HA, 44 received HA alone, 45 received PRP alone and 47 received saline. At 3, 6, and 12 months, the PRP, HA, and PRP plus HA groups had statistically significant improvements in pain relief and shoulder function compared to the saline group. At 1 and 3 months, the PRP plus HA group and the HA group had less pain than the PRP group, but the difference was not statistically significant. At the 6- and 12-month follow-up assessments, the PRP plus HA group demonstrated significantly superior pain relief and functional improvement compared to the PRP group, and the PRP group had significantly greater pain and functional improvement than the HA group.

Two RCTs have compared the efficacy of HA and CSIs for rotator cuff tendinopathy. Kim et al.^25^ compared three weekly USG injections of HMW HA in the subacromial bursa with a single CSI for rotator cuff tendinopathy without tears. They recommended rotator cuff strengthening exercises to all participants of both groups who were still symptomatic at the 3-week follow-up. Both the HA and glucocorticoid groups had statistically significant pain improvements at the 3, 6, and 12 weeks follow-up evaluations. At 12 weeks, the HA group showed a statistically significant difference in pain improvement over the glucocorticoid group.

Penning et al.^26^ also compared the effect of HA versus CSIs in patients with symptomatic subacromial impingement, who were recruited solely based on a positive painful arc and without imaging. Participants received three weekly landmark-guided subacromial injections of MMW HA, glucocorticoids, or saline. At 6 and 12 weeks from the first injections, the glucocorticoid and saline groups had statistically greater pain and function improvement than the HA group. At 26 weeks, all three groups showed significant improvement in pain and function compared to baseline, but there was no statistically significant difference between the groups.

Two additional RCTs on rotator cuff tendinopathy assessed the efficacy of HA compared with saline injection. Moghtaderi et al.^27^ compared the efficacy of three weekly USG injections of MMW HA versus three weekly saline injections in the subacromial bursa in patients with partial rotator cuff tears. They did not distinguish if the partial tears were located on the bursal or articular side. The HA group demonstrated statistically greater pain and functional improvement than the saline group at the 12-week assessment.

Chou et al.^28^ compared five weekly landmark-guided LMW HA injections in the subacromial space against five weekly saline injections. Among their participants, 33% had articular-sided tears, 9% had bursal-sided tears, and 58% had tendinopathy without a tendon tear. They found statistically greater pain and functional improvement in the HA group at six weeks post-treatment but did not perform a subgroup analysis to determine if a particular type of partial tear responded better. While both the HA and saline groups showed significant pain and functional improvement compared to baseline, the HA group showed statistically significant pain improvement over the saline group at 6 weeks follow-up from the final injection.

Esmaily et al.^29^ led an RCT on individuals with rotator cuff tendinopathy. Participants were randomized into three groups: a single LMW-HA injection, a single HMW-HA injection, or physical therapy alone. The subacromial HA injections were administered using USG. HA injections were more effective than physical therapy in reducing pain and improving range of motion and function at 3 months, with no significant difference between the LMW and HMW groups.

Rezasoltani et al.^30^ compared one USG LMW HA injection versus three weekly physical therapy sessions for 12 weeks, which included transcutaneous electrical nerve stimulation, pulsed ultrasound, stretching, and strengthening exercises of the shoulder muscles. The participants in the HA group were asked to remain active but to avoid strenuous activities following the HA injection. Their results showed that both groups experienced significant pain and function improvement compared to baseline. The participants in the HA group had a significantly greater pain improvement and quality of life improvement (physical and psychological domains) at 12 weeks than those in the PT group. As for function improvement, the difference was not statistically significant in the DASH questionnaire, but the PT group had a significantly greater improvement in the “Sports-Art” subcategory. No participants reported an adverse event.

Ko et al.^31^ compared the efficacy of three weekly landmark-guided subacromial LMW HA injections alone versus three HA injections followed by one focused extracorporeal shockwave therapy (F-ESWT) session right after the HA injection versus the same three HA injections followed by two ESWT sessions. All three groups had significant pain and functional improvement from baseline through 12 months. There was no significant difference in pain improvement between the three groups. However, functional improvement at 3 and 6 months was greater in both HA + F-ESWT groups compared to HA alone, but not significantly different at 12 months. Also, there was no significant difference in pain or functional improvement between the groups that received one and two ESWT sessions throughout the study. No participant reported an adverse event.

Flores et al.^32^ assessed the efficacy of two weekly USG MMW subacromial HA injections combined with physical therapy versus physical therapy without injections. Physical therapy for both groups in this study was performed three times per week. Both groups had significant pain and functional improvement at 30 and 90 days compared to baseline. The group with HA combined with physical therapy had a slightly greater improvement in pain than the physical therapy group throughout the follow-ups, but the difference was not statistically significant. In the HA group, 9.5% (4/42) of participants reported increased shoulder pain in the short-term period following the HA injection.

Merolla et al.^33^ led a prospective non-randomized study on the effect of two weekly USG LMW subacromial HA injections compared to three weekly physical therapy sessions during 30 days that included stretching exercises, scapula stabilization, and strengthening exercises of the shoulder muscles. By the 4-week and the 12-week marks, the HA group had a significantly greater pain improvement than the physical therapy group. At 24 weeks there was no significant improvement from baseline in either group. None of the participants had an adverse event.

Cipolleta et al.^34^ observed the effect of two USG LMW HA into the subacromial bursa 2 weeks apart in participants with supraspinatus tendon partial tear or small full-thickness tear, without a control group. The participants were asked to rest for one week after each injection followed by a progressive home exercise program. Two weeks after the first injection, there was significant pain and functional improvement. At 12 weeks, pain and function further improved, but this difference was not statistically significant compared to the two-week mark.

### Influence of MW

Two randomized-controlled trials (RCTs) have evaluated the efficacy of HA injections with different MWs for rotator cuff tendinopathy specifically. Mohebbi et al.^35^ enrolled 56 participants with rotator cuff tendinopathy and excluded only those with full-thickness tears. They did not distinguish participants who had partial tears versus those who had no tears. The included participants received a single 2 mL USG HA injection of either 500–700 kDa (LMW) or >2000 kDa (MMW or HMW) HA in the subacromial bursa. Both LMW and MMW/HMW groups showed similar improvements in shoulder pain, range of motion, and quality of life up to 6 months post-injection, with no statistically significant distinction between the two groups. However, pain at the site of the injection was more prominent in the MMH/HMW group compared to the LMW group.

In another RCT mentioned above, Esmaily et al.^29^ compared the effect of two different MWs of HA. The first group received a 500–700 kDa HA injection while the second received a  > 2000 kDa HA injection, and there was no significant difference in pain or function evolution between the two groups. However, similar to the study by Mohebbi et al., participants in the HMW group had more tenderness at the injection site than participants in the LMW group.

### Number of HA injections

The number of HA injections for rotator cuff tendinopathy ranges from one to five in the studies included in this review, and the average is 2.7 injections per participant. Most studies performed HA injections with a one-week interval between each, while two studies had a two-week span, and one study had a three-week span between each injection. No study has compared the effect of different numbers of HA injections on rotator cuff tendinopathy.

### Injection approach and guidance

The subacromial bursa is the most common HA injection site for rotator cuff tendinopathy,^36^ because the subacromial bursa is adjacent to three of the four rotator cuff tendons.^37^ A systematic review by Daley et al. reported a higher accuracy rate (100%) with ultrasound guidance than landmark guidance (72%) regarding subacromial injections.^38^

In this review, among the eight RCTs that assessed the efficacy of HA versus other treatments for rotator cuff tendinopathy, six used ultrasound guidance, and two used landmark guidance. Five out of six studies (83%) using ultrasound guidance showed favorable outcomes, whereas only one of the two studies (50%) using landmark guidance came up with outcomes in favor of HA. No study compared the efficacy of HA injections for rotator cuff tendinopathy using ultrasound guidance versus landmark guidance.

#### Combination with other rehabilitation modalities

One study combined HA injections with ESWT as part of the rehabilitation protocol. Indeed, Ko et al. had participants do one or two F-ESWT sessions right after the HA injections, which led to outcomes favoring the combination of HA and F-ESWT. No study on rotator cuff tendinopathy has yet combined HA injections and physical therapy exercises compared to HA injections alone to assess the plus value of prescribing rehabilitation exercises following an HA injection for rotator cuff tendinopathy.

## Discussion

Based on the included studies in this review, HA injections provide pain and functional improvement in rotator cuff tendinopathy. Compared to saline, three of four studies showed favorable results up to a year post-injection.^24,27,28^ There is conflicting evidence regarding the difference in the efficacy of HA versus CSI. Among the two RCTs that compared HA and CSI for rotator cuff tendinopathy, the more rigorously designed RCT by Kim et al.^25^ found a significantly greater pain and function improvement with HA injection for the first 3 months. The only study comparing HA and PRP injections showed that the best results at 6 and 12 months are obtained when PRP and HA are combined, followed by PRP alone, and finally, HA alone.^24^

Three studies showed that HA injections were superior to physical therapy at 3 and 6 months for rotator cuff tendinopathy for pain and functional improvement, while one study suggested that HA injections combined with physical therapy were equivalent to physical therapy alone.^29,30,32,33^

Linear HAs of different molecular weights have been assessed, but no study has evaluated the efficacy of cross-linked HA for rotator cuff tendinopathy. While relying on limited data, the two RCTs that specifically compared different molecular weights of HA injections for rotator cuff tendinopathy demonstrated that the molecular weight of HA did not significantly impact the degree of improvement of pain, range of motion, or quality of life.^29,35^ However, LMW-HA seems to induce milder adverse effects at the injection site, such as less pain.

No study has assessed the influence of the number of HA injections on the treatment response in rotator cuff tendinopathy. While various numbers and timing of injections are utilized in the studies, there is no evidence to support one protocol over another.

Ultrasound guidance provides greater accuracy for a subacromial injection. Furthermore, favorable outcomes compared with those who used landmark guidance, as shown in Table 1. Moreover, HA is known to cause tendinous deterioration when injected into the Achilles tendons of rats,^39^ results that raise the importance of injection accuracy with HA.

Of all studies of HA in rotator cuff tendinopathy, no participant experienced a complication or adverse effect other than transient pain at the injection site. Compared to CSIs, HA injections do not cause systemic adverse effects nor any detrimental effect on the tendon tissue, making it a safer alternative to corticosteroids.

Another treatment that seems to have a synergistic effect with subacromial HA injections is the F-ESWT applied on the rotator cuff tendons. F-ESWT is considered an effective treatment for rotator cuff tendinopathy,^40^ and studies show that combining HA injections with F-ESWT yields better results than HA injections alone or F-ESWT alone. Those findings align with previously published clinical guidelines for rotator cuff tendinopathy, revealing that a multidisciplinary and multimodal rehabilitation approach may be beneficial.^41^

## Conclusion

HA injections improve pain and function in rotator cuff tendinopathy and serve as a safer alternative to CSIs. Thus far, LMW HA, MMW, and HMW-HA show equivalent efficacy in rotator cuff tendinopathy, with LMW-HA causing the least post-injection soreness. Ultrasound guidance seems to be the most accurate guidance modality for the injections of HA into the subacromial bursa. The optimal number of HA injections and the optimal post-HA injection rehabilitation protocol are yet to be determined. Considering that HA injections have promising effects on rotator cuff tendinopathy, it is crucial for patients, clinicians, and stakeholders to optimize those therapies’ variables as much as possible.
